# Electronic Cigarette Users' Perspective on the COVID-19 Pandemic: Observational Study Using Twitter Data

**DOI:** 10.2196/24859

**Published:** 2021-01-05

**Authors:** Yankun Gao, Zidian Xie, Dongmei Li

**Affiliations:** 1 Department of Clinical & Translational Research University of Rochester Medical Center Rochester, NY United States

**Keywords:** COVID-19, Twitter, infodemiology, Electronic cigarette, perspective, observational, social media, vulnerable, sentiment analysis, topic modeling, concern

## Abstract

**Background:**

Previous studies have shown that electronic cigarette (e-cigarette) users might be more vulnerable to COVID-19 infection and could develop more severe symptoms if they contract the disease owing to their impaired immune responses to viral infections. Social media platforms such as Twitter have been widely used by individuals worldwide to express their responses to the current COVID-19 pandemic.

**Objective:**

In this study, we aimed to examine the longitudinal changes in the attitudes of Twitter users who used e-cigarettes toward the COVID-19 pandemic, as well as compare differences in attitudes between e-cigarette users and nonusers based on Twitter data.

**Methods:**

The study dataset containing COVID-19–related Twitter posts (tweets) posted between March 5 and April 3, 2020, was collected using a Twitter streaming application programming interface with COVID-19–related keywords. Twitter users were classified into two groups: Ecig group, including users who did not have commercial accounts but posted e-cigarette–related tweets between May 2019 and August 2019, and non-Ecig group, including users who did not post any e-cigarette–related tweets. Sentiment analysis was performed to compare sentiment scores towards the COVID-19 pandemic between both groups and determine whether the sentiment expressed was positive, negative, or neutral. Topic modeling was performed to compare the main topics discussed between the groups.

**Results:**

The US COVID-19 dataset consisted of 4,500,248 COVID-19–related tweets collected from 187,399 unique Twitter users in the Ecig group and 11,479,773 COVID-19–related tweets collected from 2,511,659 unique Twitter users in the non-Ecig group. Sentiment analysis showed that Ecig group users had more negative sentiment scores than non-Ecig group users. Results from topic modeling indicated that Ecig group users had more concerns about deaths due to COVID-19, whereas non-Ecig group users cared more about the government’s responses to the COVID-19 pandemic.

**Conclusions:**

Our findings show that Twitter users who tweeted about e-cigarettes had more concerns about the COVID-19 pandemic. These findings can inform public health practitioners to use social media platforms such as Twitter for timely monitoring of public responses to the COVID-19 pandemic and educating and encouraging current e-cigarette users to quit vaping to minimize the risks associated with COVID-19.

## Introduction

The World health Organization declared COVID-19 as a pandemic on March 11, 2020 [1]. The United States has reported the highest number of confirmed COVID-19 cases globally [2]. With the spread of COVID-19, significant concern has been raised about the potential increased risk for electronic cigarette (e-cigarette) users to COVID-19 infection and related mortality [3,4]. Recent studies have shown that nicotine increases the expression of the angiotensin-converting enzyme 2 (ACE-2) in human bronchial epithelial cells. ACE-2 is the binding site for SARS-CoV-2, the virus that causes COVID-19 [5-8]. A national online survey study of 4351 youth and young adults showed a 5-fold increase in COVID-19 diagnoses among ever e-cigarette users compared to non-users [9]. However, no study has evaluated the attitudes of e-cigarette users toward the COVID-19 pandemic and whether their attitudes differ from those of nonusers. Therefore, it is important to characterize how e-cigarette users perceive the COVID-19 pandemic and how their perception differs from that of non-users. These findings will facilitate us to understand how e-cigarette users might respond to the COVID-19 pandemic, especially in terms of vaping.

Twitter is one of the most popular social media platforms, with an average of 330 million monthly active users sharing content on the platform, as of 2019 [10]. Twitter users can publish publicly available posts (called tweets), making Twitter a rich data source to monitor social phenomena and public health issues [11]. This study focused on understanding how Twitter users in the United States who used e-cigarettes responded to the COVID-19 pandemic by using sentiment analysis and topic modeling to extract users’ subjective attitudes and to identify topics from the textual contents of their tweets. Understanding the attitudes of e-cigarette users toward the COVID-19 pandemic and topics discussed by them on Twitter could help public health workers and policymakers take appropriate actions such as encouraging e-cigarette users to quit vaping during the current COVID-19 pandemic.

## Methods

### Data Collection

Since the correlation between COVID-19 and e-cigarettes has been a popular topic during the current pandemic, tweets about e-cigarettes in our COVID-19 dataset were not necessarily from e-cigarette–related user accounts. Therefore, to identify e-cigarette users, we used an e-cigarette–related dataset from 2019, that is, before the COVID-19 pandemic. Tweets were collected between May 2019 and August 2019 through a Twitter streaming application programming interface (API) by using e-cigarette–related keywords (ie, “e-cig,” “e-cigs,” “Ecig,” “Ecigs,” “electroniccigarette,” “Ecigarette,” “Ecigarettes,” “vape,” “vapers,” “vaping,” “vapes,” “e-liquid,” “ejuice,” “eliquid,” “e-juice,” “vapercon,” “vapeon,” “vapefam,” “vapenation,” and “juul”) [12,13]. In addition, a list of spam-specific keywords was used to remove tweets that were unrelated to e-cigarettes [14]. In this e-cigarette–related dataset, Twitter users whose username and user screen name did not contain any e-cigarette keywords were considered as e-cigarette users. Although we intended to use tweets before the announcement of flavor ban policies in different states (starting from September 2019) to identify users who tweeted about e-cigarettes in order to avoid the potential noise, the starting point (ie, May 2019) was randomly selected.

The COVID-19 dataset was collected using a Twitter streaming API to crawl Twitter posts between March 5, 2020, and April 3, 2020, with coronavirus-related keywords (“CORONA,” “corona,” “COVID19,” “covid19,” “covid,” “coronavirus,” “Coronavirus,” “CoronaVirus,” and “NCOV”), which were identified from COVID-19–related tweets. Twitter IDs were used to identify unique Twitter users. To get a clean dataset, promotion-related Twitter IDs and posts were filtered out. In addition, tweets that mentioned “corona” (a brand name for beer) as a beverage were removed from the dataset. The keywords used to clean the COVID-19 dataset included “dealer,” “deal,” “supply,” “beer,” “drink,” “drank,” “drunk,” “store,” “promo,” “promotion,” “customer,” “discount,” “sale,” “free shipping,” “sell,” “$,” “%,” “dollar,” “offer,” “percent off,” “save,” “price,” and “wholesale”. After filtering the data, US-based Twitter posts were selected using geolocation keywords, such as “United States,” “New York,” “USA,” and “US.” Duplicate tweets were removed, and retweets were included in the final dataset. The tweets in the US COVID-19 dataset, which were posted by the above-identified e-cigarette users, were classified as the e-cigarette (Ecig) group. The remaining COVID-19 tweets were classified as the non-e-cigarette (non-Ecig) group.

### Ethical Statement

In this descriptive, observational study, we collected and analyzed user-generated content from Twitter. No intervention or interaction was made with the users who posted information on Twitter. The identifiers that could be associated with the Twitter data are usernames or Twitter handles, which are accessible by the public or anyone with internet access. All the usernames or Twitter handles in the study were randomly assigned a numerical number after the Twitter data was collected.

### Data Availability Statement

The data and scripts used for the analyses and to create figures are available on request from the corresponding author.

### Sentiment Analysis

Sentiment analysis refers to the contextual mining of an incoming message, which can extract the underlying attitudes and determine whether the sentiment is positive, negative, or neutral. The sentiment score for each tweet in our dataset was computed using VADER (Valence Aware Dictionary and sEntiment Reasoner), a tool used to obtain sentiments from social media data [15]. Each user’s average sentiment score was calculated for each day of the study period. The mean value of the average sentiment score of users from the same group was then calculated to represent the overall group sentiment for each day. A sentiment score of +0.05 or higher denotes a positive attitude. A sentiment score −0.05 or lower denotes a negative attitude. A sentiment score between −0.05 and +0.05 denotes a neutral attitude. The mean sentiment scores were then examined longitudinally across the study period to evaluate their potential links with COVID-19 spread and government policy changes.

### Topic Modeling

Topic modeling, specifically the latent Dirichlet allocation (LDA) model, was used for text content analysis. LDA is a 3-layer hierarchical Bayesian model, in which each word in the document is modeled into a specific topic, and the words in each topic are weighted based on their appearance [16]. Using the LDA model allowed us to identify the topics of conversations in both Ecig and non-Ecig groups. Next, data cleaning processes were performed. All punctuation, white spaces, stop words were removed. In addition, uppercase characters were converted to lowercase characters. Words were lemmatized to their stem form to ignore different tenses, and frequent bigrams and trigrams were identified using a Python library, Gensim. Topic modeling was applied to tweets from both Ecig and non-Ecig groups. Topic coherence was used to determine the optimal number of topics to identify the frequently discussed topics in each group [17].

## Results

The US COVID-19 tweets dataset between March 5, 2020, and April 3, 2020, consisted of 10,902,142 tweets from 2,144,599 unique Twitter users. From the e-cigarette–related tweets dataset generated between May 2019 and August 2019, we identified 930,290 tweets from 902,310 unique Twitter users. From the COVID-19 tweets collected, we identified 11,479,773 tweets from 2,511,659 unique Twitter users in the non-Ecig group and 4,500,248 tweets from 187,399 unique Twitter users in the Ecig group.

**Figure 1:**
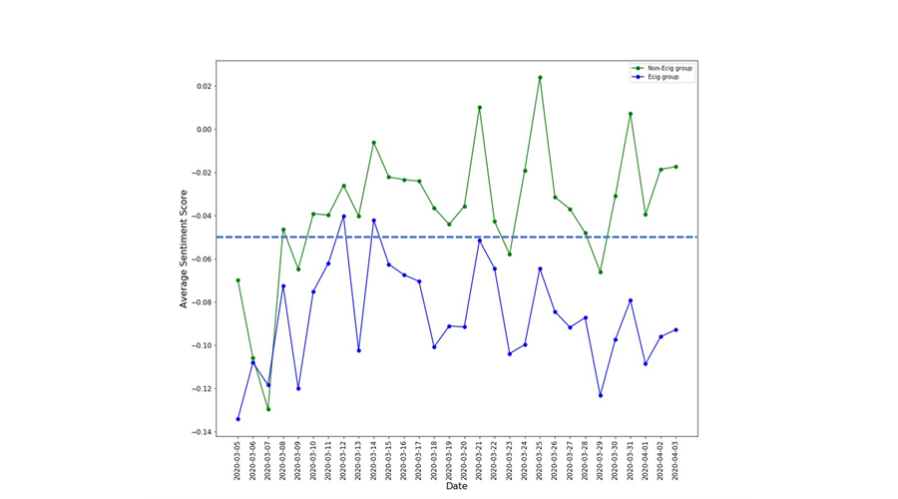
Comparison of different sentiments toward COVID-19 between US-based Twitter users who used e-cigarettes (Ecig group) and those who did not use e-cigarettes (non-Ecig group) from March 5, 2020, to April 3, 2020.

Figure 1 shows the average sentiment score of COVID-19 tweets in each group from March 5 to April 3, 2020. Users in neither the Ecig group nor the Non-Ecig group showed a positive attitude. Other than on March 7, 2020, Ecig group users showed a more negative attitude towards COVID-19 than non-Ecig group users. Except in early March, the average sentiment scores of non-Ecig group users were mostly neutral. In contrast, Ecig group users had a negative sentiment for almost the entire study period. The sentiment scores from both groups showed similar trends over time.

To obtain content-wise insights from the discussions in Ecig and non-Ecig groups, the LDA topic model was applied to the tweets posted by users from both groups. Tables 1 and 2 summarize the popular topics discussed in each group, including the top 10 keywords for each topic. The top 3 topics (percentage of tokens) in the Ecig group included “Trump handling corona” (12.8%), “Death toll” (11.7%), and “Stay home” (11.3%). The top 3 topics (percentage of tokens) in the non-Ecig group included “Trump blame China” (12.9%), “Hospital caring and testing” (10.7%), and “COVID testing” (10.5%).

**Table 1:** Major topics discussed based on COVID-19 tweets posted by e-cigarette users (Ecig group) in the US, from March 5, 2020, to April 3, 2020.

Topic	Percentage of tokens (%)	Keywords
Trump handling corona	12.8	Trump, say, call, lie, take, would, president, response, medium, job
Death toll	11.7	Test, case, death, positive, new, report, number, confirm, people, day
Stay home	11.3	Health, home, stay, pay, public, worker, family, leave, need, emergency
Death and virus spread	10.7	Virus, corona, people, take, year, many, know, die, spread, time
Testing of COVID	9.3	Get, test, day, week, say, kit, testing, go, covid, make
Trump wants hospitals and doctors to help patients	9.2	Patient, trump, want, help, people, doctor, know, give, say, hospital
Virus spread	9.1	Spread, Chinese, stop, response, virus, help, make, pandemic, covid, global
Combat with COVID	7.1	Know, vote, say, hand, bill, would, may, travel, good, time
School shutdown	7	Due, close, cancel, go, concern, hear, school, people, get, say

**Table 2:** Major topics discussed in COVID-19 tweets posted by e-cigarette nonusers (non-Ecig group) in the US, from March 5, 2020, to April 3, 2020.

Topic	Percentage of Tokens (%)	Keywords
Trump blame China	12.9	Trump, say, call, people, lie, Chinese, medium, government, response, crisis
Hospital caring and testing	10.7	Covid, need, patient, hospital, fight, help, care, worker, testing, family
COVID testing	10.5	Virus, corona, test, get, positive, people, symptom, day, know, go
Death toll	10.4	Case, death, report, number, test, new, confirm, day, first, state
Stop work and stay home	9.6	People, work, amp, stop, hand, go, die, stay, home, get
School and business shutdown	9	Due, spread, school, cancel, close, concern, health, public, plan, business
Relief bill	8.1	Vote, watch, time, hold, people, bill, play, run, relief, help
Stay home	8	Take, home, stay, covid, go, people, order, spread, good, say
Response to COVID	7.9	Thank, response, question, covid, share, late, update, ask, release, amp

## Discussion

### Principal Findings

During the COVID-19 pandemic, people worldwide have widely used Twitter to follow news and express their opinions and responses to the pandemic [18]. Although Twitter users in the non-Ecig group had a neutral attitude toward COVID-19 during most of the study period (March 5 to April 3, 2020), Twitter users in the Ecig group had a negative attitude toward this pandemic. The topics most frequently discussed by Ecig group users were how the US President Donald Trump handled COVID-19, deaths due to COVID-19, and staying at home. On the other hand, the most frequently discussed topics in the non-Ecig group included Trump blames China, hospital care for patients with COVID-19, and COVID-19 testing. The differences between Ecig and non-Ecig group users’ attitudes toward the COVID-19 pandemic indicated a good opportunity to educate e-cigarette users about the potential harms of vaping and encourage them to quit vaping during the COVID-19 pandemic.

The average sentiments of users from both Ecig and non-Ecig groups were relatively parallel during the study period, which suggests that the dynamic changes of the COVID-19 pandemic and other factors (such as government policies) had similar effects on the sentiments of e-cigarette users and nonusers towards the COVID-19 pandemic. We noticed, however, that some variation in the sentiment scores might be associated with the government’s policies. For example, when all nonessential businesses in New York City—the worst-affected area—were closed on March 22, 2020, sentiment scores of users in both Ecig and non-Ecig groups decreased to trough on March 23. The sentiment scores of non-Ecig group users quickly reached the highest peak on March 25 when the Congress agreed on a $2 trillion virus relief package bill.

Our study findings show that Ecig group users presented a more negative attitude towards the COVID-19 pandemic than did non-Ecig group users. Moreover, Ecig group users discussed more topics related to death and virus spread. Some of the common topics discussed in both groups included how Trump responded to COVID-19, deaths due to COVID-19, and social distancing practices such as staying at home and shutting down schools. One of the top topics unique to the discussion in the Ecig group was death and virus spread, which did not feature among the top topics discussed in the non-Ecig group. The concerns in the Ecig group about the virus spread and COVID-19–related deaths might be related to the discussions that vaping may increase the risk of severe COVID-19 infection. Starting from 2019, the epidemic of vaping associated lung injury (EVALI) in the US drew significant attention among the public [19]. An early study (February 2020) showed that patients with COVID-19 had similar symptoms as EVALI, such as fever and cough, as well as characteristic lung phenotypes [20]. In addition, studies have shown that e-cigarette use can suppress the genes related to the immune and inflammatory response [21,22], which could increase the duration and severity of respiratory infections. These findings might lead to more concerns about the possible connection between vaping and the COVID-19 pandemic for e-cigarette users.

Systematic surveillance of vaping-related discussions on Twitter identified public health–related topics at the intersection of vaping and COVID-19; these topics included health concerns as well as unsubstantiated health claims [23]. Currently, there is a lack of evidence that e-cigarette users are more susceptible to COVID-19 infection and death. Although public health experts claim that vaping and smoking could increase the risk of COVID-19 infection, and multiple research studies have suggested that smoking is associated with adverse outcomes of COVID-19 [24,25], a study in Europe published contrasting conclusions that daily smokers had a lower risk of developing severe COVID-19 symptoms [26]. Future studies should further investigate the association of smoking or vaping with COVID-19 infection and death. Notably, the abovementioned European study was published in the end of April 2020, which was beyond our study period. Therefore, how the report of the negative association between smoking and COVID-19 affects the sentiments of people, especially e-cigarette users and smokers, awaits further investigation.

### Limitations

This study has several limitations. First, as with many other social media studies using Twitter data, significant geographic bias exists in the sentiments expressed in tweets over the same time period [27]. Moreover, the sentiments expressed in tweets could be biased based on the geographic location—whether the user is local or visiting that area and what other activities they have completed during the course of a day [28]. Second, the generalization of the study results is limited by the representation of Twitter users in the general population. Twitter users are relatively younger and more educated than the general population [29]. Highly active Twitter users also have different behaviors than the rest of the Twitter population [29]. Third, some Twitter account types, such as information aggregators, which could also aggregate vaping discount information but were not e-cigarette users, were not removed from our dataset and could introduce some bias in the results of the analysis [30]. Furthermore, the non-Ecig group may include some e-cigarette users who were not identified from the earlier e-cigarette–related dataset, which could also introduce bias in the results. Fourth, some Twitter accounts were marked as private from the API; therefore, we were unable to retrieve tweets from those accounts. Fifth, only a small proportion of Twitter accounts provided the geolocation, and we could only select Twitter accounts that provided this information [31]. Sixth, other than human users, there are some social bots accounts on Twitter. However, those bot accounts were not excluded in this study, which may also cause some bias. Moreover, this study did not identify smokers in both groups who might have different attitudes towards the pandemic, which might lead to some additional bias in the results. As we defined the Ecig group based on Twitter data collected from May to August 2019, e-cigarette users who did not post e-cigarette–related tweets during this period might be mislabeled and subsequently misclassified into the non-Ecig group. Moreover, non-Ecig users in that period could have become e-cigarette users during the COVID-19 pandemic. This could introduce potential selection bias and misclassification in both directions given the time lag. Seventh, we could not distinguish individual accounts from institutional or group accounts based on the Twitter data; thus, the information about user attitudes toward COVID-19 might not all represent individuals. Finally, our study period was in the early stage of the pandemic with limited information available about the potential link between vaping and COVID-19, which might introduce some biases. With the rapid spread of the COVID-19 pandemic and emergence of more evidence on the link between vaping and COVID-19, the perception and responses to the COVID-19 pandemic of the public, including e-cigarette users, might evolve; however, this requires re-evaluation of the outcomes using more recent Twitter data.

### Conclusions

In this study, Twitter users in the Ecig group showed a more negative attitude toward the COVID-19 pandemic than those in the non-Ecig group. This study highlights the importance of using Twitter for surveillance of public responses to the COVID-19 pandemic, which can provide early insights for public health awareness, especially among specific population groups (such as e-cigarette users). Users in the Ecig group discussed topics such as the spread of the virus and COVID-19–related deaths, which highlights these vapers’ concerns about the potentially elevated risks of COVID-19. These findings may provide a useful opportunity for public health practitioners to educate current e-cigarette users and encourage them to quit vaping to reduce the risks associated with COVID-19.

### Authors’ Contributors

YG, ZX, and DL conceived and designed the study. YG analyzed the data. YG wrote the manuscript. YG, ZX, and DL assisted with the interpretation of analyses and edited the manuscript.

**Conflicts of Interest**

None declared.

## Abbreviations

ACE-2: angiotensin-converting enzyme 2
API: application programming interface
Ecig users: electronic cigarette users
EVALI: epidemic of vaping associated lung injury
LDA: latent Dirichlet allocation
Non-Ecig users: non-electronic cigarette users
VADER: Valence Aware Dictionary and sEntiment Reasoner
